# Dominant Cultural and Personal Stigma Beliefs and the Utilization of Mental Health Services: A Cross-National Comparison

**DOI:** 10.3389/fsoc.2019.00040

**Published:** 2019-05-08

**Authors:** Piet Bracke, Katrijn Delaruelle, Mieke Verhaeghe

**Affiliations:** ^1^Department of Sociology, Ghent University, Ghent, Belgium; ^2^Department of Applied Social Studies, VIVES University of Applied Sciences, Kortrijk, Belgium

**Keywords:** cultural stigma beliefs, personal stigma beliefs, mental health, professional-care seeking, cross-national comparison

## Abstract

**Purpose:** The detrimental impact of stigma on the utilization of mental health services is a well-established finding. Nevertheless, most studies consider only the personal or interactional dimensions of stigma. This contribution makes a distinction between the dominant beliefs about stigma within a culture and the personal beliefs of individuals with regard to stigma. We hypothesize that both have an impact on professional-care seeking within the field of mental health.

**Methods:** A multi-level research design is used to estimate the effects of both types of stigma beliefs on the likelihood of consulting general and specialized health professionals about mental health problems in 28 European countries (N of individuals = 24,881, Eurobarometer 248, 2005–2006).

**Results:** In countries where stigmatizing beliefs are dominant, the likelihood of seeking help from specialized mental health professionals is constrained, and individuals refrain from contacting general practitioners when in need of formal support, regardless of their own personal stigma beliefs.

**Conclusion:** The present study signals the importance of stigma beliefs as shared cultural phenomena, and of personal stigma beliefs to the likelihood of seeking professional care for mental health problems. We therefore propose that most studies on stigma and formal-care seeking underestimate the pervasive effects of stigma beliefs, due to methodological individualism.

## Introduction

Public-opinion studies have confirmed the widespread existence of negative stereotypes about individuals with mental health problems: they are considered dangerous, unpredictable, and difficult to talk to Pescosolido et al. (1999). These negative stereotypes can have detrimental consequences on the users of mental health services through processes of public stigma (Angermeyer and Matschinger, 2005), self-stigma (Corrigan and Watson, 2002), and stigma expectations (Link et al., 1989) with regard to such outcomes as self-efficacy (Markowitz, 1998), depressive symptoms (Link et al., 1997), self-esteem (Link et al., 2001; Verhaeghe et al., 2008), life satisfaction (Rosenfield, 1997), client satisfaction, work, and income (Link, 1982), and social relationships (Prince and Prince, 2002), in addition to treatment continuation and medication adherence (Sirey et al., 2001). For a comprehensive overview, see the studies by Sickel et al. (2014), Clement et al. (2015), and Schnyder et al. (2017). One common conclusion of such studies is that stigma diminishes the beneficial effects of mental health treatment (Rosenfield, 1997), in addition to inducing negative consequences at an earlier stage of the process of seeking professional help. Individuals whose perceptions or attitudes concerning mental illness are more stigmatizing tend to be less likely to seek professional mental health care (Barney et al., 2006; Vogel et al., 2007). Stigma thus constitutes a major barrier to the utilization of mental health services (Corrigan, 2004; Ojeda and Bergstresser, 2008).

The notion of stigma as a phenomenon that plays out within a social context or culture is prevalent in early treatises on the stigma of mental illness (Goffman, 1963), as well as in more recent works (Link and Phelan, 2001; Yang et al., 2007; Pescosolido et al., 2008; Corrigan et al., 2014; Hatzenbuehler and Link, 2014; Phelan et al., 2014; Hatzenbuehler, 2016). These works emphasize the macro-social dimension of the scourge. Hatzenbuehler and Link (2014) define the construct of “structural stigma” as “*societal-level conditions, cultural norms, and institutional policies that constrain the opportunities, resources, and well-being of the stigmatized*” (2014, p. 2). One component of structural stigma thus consists of the negative attitudes and stereotypes that members of a community share with regard to individuals with mental health problems. Link and Phelan (2001) refer to this component of structural stigma as the cultural conceptions of mental illness or “dominant cultural beliefs.” They further state that the power of stigma emerges within a cultural system as a “set of cognitive and evaluative beliefs—beliefs about what is or what ought to be—that are shared by the members of a social system and transmitted to new members” (House, 1981, p. 542) cited in Link and Phelan (2014). These shared beliefs differ from an individual's negative attitudes and stereotypes about persons with mental health problems, called personal stigma beliefs (Nearchou et al., 2018). Pescosolido et al. (2008) explicitly refer to both the individual and the contextual influences of stigma. Following Steele (1997), they highlight the pervasive nature of stereotypes held by others in society and the relevance of the societal context. Yang et al. (2007) also situates stigma unequivocally at the cultural level. In the present study, we take this cultural context into account when investigating the link between mental health stigma and the seeking of professional mental health care. Drawing on Link and Phelan (2001), we draw a conceptual distinction between the concept of dominant cultural stigma beliefs and personal stigma beliefs, using a cross-national comparative multi-level design to estimate their independent effects.

The consideration of stigma as a cultural phenomenon that differs across countries has several conceptual implications. First, it implies that citizens of the same country or members of the same culture share stigmatizing perceptions and attitudes at least to some extent. Scholars have indeed found evidence of substantial cultural and/or cross-national differences in the prevalence of stereotypes (Beck et al., 2003; Angermeyer et al., 2004, 2005; Schomerus et al., 2006, 2015; Yang et al., 2007; Haraguchi et al., 2009; Mojtabai, 2010; Pescosolido, 2013). Second, not all citizens of a given country necessarily share such beliefs to the same extent: there might be little or no correspondence between dominant cultural beliefs about stigma and personal beliefs in this regard. For example, one could be well-aware of the dominant stigma beliefs without adhering to them personally (Corrigan and Watson, 2002). Alternatively, the discrepancy between personal and dominant cultural stigma beliefs might elicit angry reactions in individuals in response to perceived prejudice (Corrigan and Watson, 2002). Although such possibilities are obvious, their methodological consequences are often ignored. For example, despite general consensus that stigma should be conceived of as a cultural phenomenon, it is usually measured at the individual level. As a result, most studies focus on the relationship between the stigmatizing attitudes of individuals and their attitudes or behaviors concerning the use of mental health services. Few if any studies have addressed the impact of structural stigma on efforts to seek professional help (Quinn and Chaudoir, 2009; Clement et al., 2015), thereby leading to the under-estimation of its total impact. Although the distinction between public stigma and self-stigma (Corrigan and Watson, 2002) has been acknowledged, public stigma is seldom measured independently of individual perceptions of public stigma (Vogel et al., 2007). At the other end of the spectrum, stigma is regarded as a collective phenomenon (Schomerus et al., 2015), completely ignoring the individual perspective. This results in the “ecological fallacy” of over-estimating the importance of prevailing cultural stigma beliefs (Robinson, 1950).

In summary, this study aims to disentangle the influence of cultural and personal stigma beliefs on efforts to seek professional help for mental health problems within a stratified sample of the adult population in 28 European countries, based on a multi-level design. We focus explicitly on one component of structural stigma (Hatzenbuehler, 2017)—the notion of stigma as a set of shared cultural beliefs (Phelan et al., 2014)—and model its impact independently of variations in personal beliefs. This approach corresponds to Corrigan's (2004) call for studies on the connection between stigma and service utilization based on research methods that incorporate both micro-level and macro-level variables.

### Stigma and the Utilization of Mental Health Services: Existing Research

Several researchers have studied the link between stigma and the utilization of mental health services in various populations using diverse conceptualizations of both stigma and service utilization. For example, in a study on the population of a rural town, Wrigley et al. (2005) found that stigma expectations were negatively associated with general attitudes toward seeking professional psychological help. In another example, Vogel et al. (2007) identifies self-stigma as a major determinant of the willingness of undergraduate students to seek counseling. In their study of the general population, Schomerus et al. (2009) demonstrate the crucial role that self-stigma plays in shaping intentions to seek psychiatric help for depression. Finally, in a prospective study based on a clinical sample, Rüsch et al. (2005) identify stigma expectations and self-stigma as predictors of service utilization.

In contrast to the findings reported above, other authors have failed to find such evidence. For example, Sareen et al. (2007) conclude that attitudinal barriers to help-seeking are far less important than structural barriers are. In a longitudinal study (Jorm et al., 2000), found that stigma expectations did not predict either future service utilization or outcomes of depression. In addition, Clement et al. (2015) conclude that the impact of stigma is at best modest. In general, however, stigma has been found to act as a barrier to seeking professional mental health care (Sickel et al., 2014; Clement et al., 2015; Schnyder et al., 2017). These findings hold regardless of how service utilization is measured (e.g., willingness to seek formal care in the future or the actual utilization of mental health services) and for various measures of stigma (e.g., self-stigma and internalized stigma; stigma expectations and anticipations; stigma experiences). Based on this evidence, we formulate our first research hypothesis at the individual level: *Individuals who adhere to stigmatizing beliefs are less likely to seek help from professionals for mental health problems than are those who do not adhere to such beliefs (H1)*.

### The Role of Cultural Stigma Beliefs: Theoretical Reasoning

The assumption that individuals' behavior is affected by the culture of the societies to which they belong is a basic tenet within the social sciences. Culture is generally conceived of as a system of shared beliefs, norms, values, and attitudes. Not all members of a community share these aspects to the same extent, however, and they can be aware of prevailing norms and values without personally adhering to them. Nevertheless, they may be affected by them, and both personal and cultural beliefs may exert interdependent, but discernible, effects. For example, with regard to mental health stigma, Link (1987); Link et al. (1989) have demonstrated that stigma expectations—“the extent to which individuals believe that ‘most people' (the community at large) will devalue and discriminate against a mental patient” (Link, 1987, p. 403)—do not differ between individuals with and without mental health problems or history of treatment. They interpret this as an indication that such expectations constitute a component of culture. In addition, stigma expectations lead individuals with mental health problems to use adaptive strategies (e.g., secrecy or withdrawal). They thus do not necessarily believe in these negative stereotypes; they only know (and fear) that they will be applied to them once they have been “labeled.” Applying the logic of Link et al. (1989), cultural stereotypes might hinder the utilization of mental health services, due to fear of discrimination and devaluation. In this vein, Wahl (1999) identifies fear of disclosure as the core reason why users of mental health services keep their psychiatric status secret. In addition, Clement et al. (2015) provide an extensive overview of the processes underlying the relationship between stigma and help-seeking for mental health problems. The preference for non-disclosure; the anticipation of social judgement, rejection, and discrimination; and the anticipation of disrespect from professionals are but a few of the many reasons why individuals refrain from seeking help, even if they do not personally adhere to such stigma beliefs. The differential effects that Schomerus et al. (2009) report that the anticipation of discrimination and personal discriminatory attitudes have on intentions to seek help can be interpreted as indirect evidence of the importance of cultural stigma. Cultural and personal stigma beliefs can also reinforce each other. For example, Vogel et al. (2007) have shown that the effect of public stigma on the utilization of mental health services operates through self-stigma. Nearchou et al. (2018) find that help-seeking intentions in adolescents are more affected by public stigma beliefs than their personal stigma beliefs.

In contrast to the aforementioned studies, we do not conceptualize cultural stigma beliefs as individual perceptions of what the “generalized other,” or “most people” believe (Pattyn et al., 2014), nor do we approach them as the perceived attitudes, beliefs, and behavioral dispositions of the general population, which are often referred to as “public stigma” (Corrigan and Shapiro, 2010; Pescosolido, 2013; Nearchou et al., 2018). The cross-national, multi-level design of our study allows us to draw a distinction between the prevailing stigma beliefs within a country and the beliefs of individuals by aggregating individuals' beliefs at the country level. Other scholars have used similar strategies, including aggregation at the level of countries (Mojtabai, 2010; Evans-Lacko et al., 2012; Pachankis et al., 2014) or schools (Gaddis et al., 2018), or within categories of stigmatized identities (Quinn and Chaudoir, 2009). Based on this reasoning, we predict that *citizens of countries with a strongly stigmatizing culture are less likely to utilize professional mental health care services, regardless of their personal stigma beliefs (H2)*.

**Figure 1:**
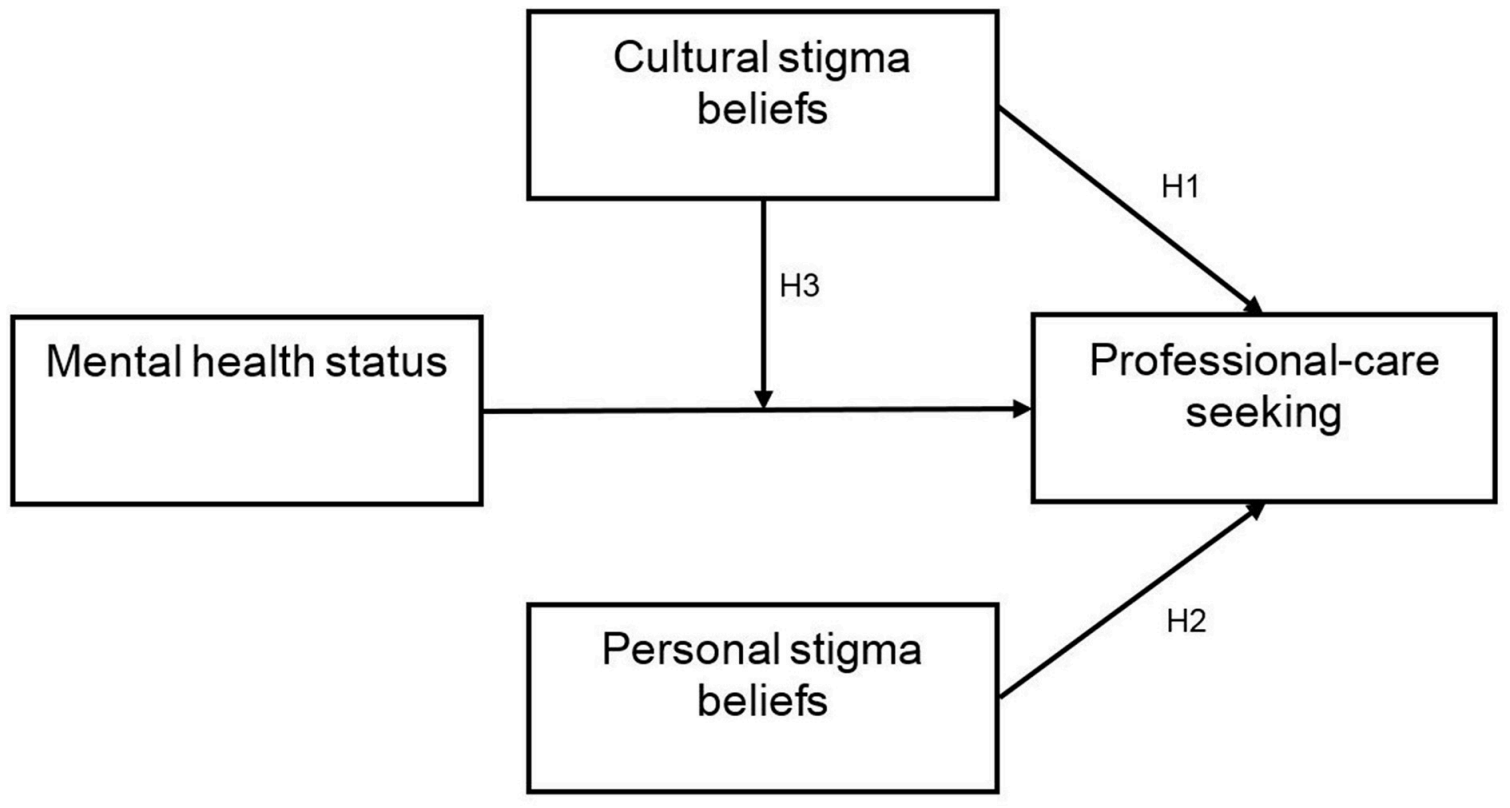
Personal and cultural stigma beliefs and professional-care seeking: Conceptual model and main hypotheses.

For purposes of clarification, the various hypotheses are visualized in Figure 1.

### Need and Supply as Determinants of the Utilization of Mental Health Services

In addition to cultural and individual stigma beliefs, we account for two other important factors determining the utilization of mental health services: societal supply of mental health services and individual need for treatment (Andersen, 1995).

Cross-national comparisons of the utilization of mental health services should consider variations in the supply of such services, as the availability and accessibility of treatment can be expected to influence utilization (Koopmans et al., 2005). Unmet need is worst in low-income and middle-income countries (Wang et al., 2007), and the treatment gap might be due to the small share of spending that these countries allocate from their already limited health budgets to the funding of mental health care (Koopmans et al., 2005). Cross-national variations in the availability and accessibility of mental health services are thus likely to influence their utilization, even in middle-income and high-income European countries. For this reason, the availability of general or more specialized professionals is a supply-side factor that should not be ignored (Saxena et al., 2007). Country-level indicators of available mental health services are nevertheless unable to capture important within-country differences in the accessibility of services. We therefore also use the degree of urbanization in the area of residence as a rough indicator of the availability of more specialized mental health professionals. Differences between rural and urban areas in the utilization of mental health services might also reflect differences in both stigma beliefs (Hoyt et al., 1997; Caracci and Mezzich, 2001) and overall mental health (Caracci and Mezzich, 2001).

With regard to the need for treatment, we assume that individuals with more frequent mental health complaints are in greater need of formal care and, all other things being equal, they tend to make more use of mental health services. Cross-national differences in unmet need have been reported (Consortium The WHO World Mental Health Survey, 2004; Wang et al., 2007), thus implying that the association between the need for mental health services and the actual utilization of services also varies between countries. We assume that cross-national differences in stigma beliefs could offer one possible explanation, as the association between the need for and the utilization of services might be attenuated in countries with more prevalent cultural stigma beliefs. Our third and final main hypothesis is that *the effect of need on the utilization of mental health services depends on the extent of societal stigma (H3)*.

## Research Strategy

The main goal of this empirical study is to investigate the link between stigma and the utilization of mental health services within the general European population. In this investigation, we use multi-level techniques to draw explicit distinctions between stigma at the individual and stigma at the cultural level. We therefore include two measures of stigma as main independent variables: personal stigma beliefs at the individual level and cultural stigma beliefs at the country level. To avoid excessive complication in the models, we ignore subtle differences in the various dimensions of stereotypes and their consequences for help-seeking behavior in the context of formal mental health services (Mojtabai, 2010). We also consider several different types of actual mental health services, given available evidence that people with stigma beliefs are more likely to avoid specialized mental health professionals (e.g., psychiatrists) than they are to avoid general practitioners (GPs) or other general health care providers (Barney et al., 2006).

The following variables are included in addition to those described above. First, age might be an important factor, as elderly people might tend to hold more stigmatizing beliefs about mental illness, and the association between poor mental health and the utilization of services is weaker for elderly people (Koopmans et al., 2005). Second, gender should be included, given the tendency of women to report more complaints related to depression and anxiety (Van de Velde et al., 2010), as well as their greater willingness to seek professional help. Moreover, some studies have suggested that men hold more stigmatizing beliefs about the utilization of mental health services (Vogel et al., 2007), and both genders apparently expect men to rely on self-care (Pattyn et al., 2015). Indicators of socio-economic status and marital status are included as well, given their association with mental health (Jenkins et al., 1997; Levecque et al., 2011) and the utilization of mental health services (Alonso et al., 2004). Because the step toward mental health service could be regarded as an information-seeking process (Vogel et al., 2006), we also include an indicator of difficulty in finding information. Such difficulties could be regarded as barriers to the utilization of mental health services, and the availability of information could be regarded as a component of service accessibility. Finally, we control for urbanization, in light of previous studies that have reported differences between rural and urban areas with regard to stigma (Economou et al., 2009), mental illness (Paykel et al., 2000) and the utilization of mental health services (Fortney et al., 1999).

## Methods

### Sample

This study is based on data collected as part of Eurobarometer 248 (2005–2006). The Eurobarometer covers the population of individuals 15 years of age and older in each of the EU Member States in 2006. In addition, Eurobarometer 248 was conducted in two countries that were then in the process of accession (Bulgaria and Romania) and two candidate countries (Croatia and Turkey), as well as in the Turkish Cypriot Community (TCC). The survey covers the national population of citizens of the respective nationalities, as well as the population of citizens of all the European Union Member States residing in those countries and have sufficient command of one of the respective national languages to answer the questionnaire. The basic sample design applied in all member states is a multi-stage, random (probability) sample of individuals within households within a specific area. All telephone interviews were conducted in the appropriate national language.

In our analyses, we merged the data from East and West Germany, from Northern Ireland and the United Kingdom, and from the Republic of Cyprus and the Turkish Cypriot Community. Because we excluded respondents 17 years of age and younger, the resulting samples are representative of the population of citizens in the included countries 18 years of and older. Due to the lack of information on the supply of mental health professionals in Estonia and to missing information on core variables for some individuals across several countries (*N* = 2422; 8.9%), the working sample was limited to 24,881 individuals in 28 countries.

### Variables

#### Individual-Level Variables

*Utilization of mental health services* or *likelihood of seeking professional help for mental health issues* was measured by responses to the question, “*In the last 12 months, did you seek help from a professional in respect of a psychological or emotional health problem?*” Respondents could select from among the following professionals: GP, pharmacist, psychiatrist, psychologist, psychoanalyst, nurse, social worker, psychotherapist not mentioned previously, and another health professional. This information was used to construct two dichotomous indicators: (1) seeking help from a GP (no = 0, yes = 1), (2) seeking help from a psychiatrist, psychologist, psychoanalyst, or psychotherapist not mentioned previously (yes on at least one of these items = 1, none = 0). We were unable to analyze the various specialized professionals separately, due to the small number of respondents reporting having consulted one or more of these professionals.

*Personal stigma beliefs* refer to adherence to negative stereotypes about individuals with mental health problems. Because the Eurobarometer contains only four items referring to stigma, our analyses and results are limited to stereotypes, which are regarded as a first, but crucial, step in the stigma process (Angermeyer and Matschinger, 2005). The items refer to the four main components of the stereotype of mental illness: dangerousness, attribution of responsibility, poor prognosis, and disruption of social interaction (Hayward and Bright, 1997; Griffiths et al., 2006). For the exact wording of the items, we refer to Mojtabai (2010). Responses range from “totally agree” (1) to “totally disagree” (4). The item scores were reversed, and mean scores were calculated for respondents with at least two valid answers. High scores indicate strong stigmatizing beliefs about people with mental health problems. The total reliability of the sample is 0.66, which is satisfactory for a four-item indicator. The overall mean score for the weighted sample is 2.18 (*SD* = 0.62).

*Mental health complaints* were measured using the short five-item version of the Mental Health Inventory (MHI-5) from the SF-36 Berwick (Berwick et al., 1991), which measures complaints relating to depression and anxiety according to such items as, “How much of the time during the past 4 weeks have you felt downhearted and depressed?” The answer categories range from “all the time” (1) to “never” (5). Negative items were reversed, with high scores indicating more frequent and more severe complaints relating to depression and anxiety. We computed scores on the instrument as a sum scale ranging from 5 to 25 for respondents who provided at least three valid answers. The psychometric characteristics of the scale are well-known and have been described in detail in other studies (McCabe et al., 1996; Rumpf et al., 2001). The total reliability score for the weighted sample is 0.83, which is high for the five-point rating-scale version of the MHI-5 (Rumpf et al., 2001). The scale measures only indicators relating to depression and anxiety. No information was available on other mental health problems.

The following control variables were included in the analysis: *gender* (female = 1, male = 0), *age* (in years) and *education*, which is measured by the respondent's age upon completing full-time education: 20 years or older, 16–19 years, and younger than 16 years (the reference category). The last category also includes those with no full-time education, and a separate category was added for respondents whose educational level is unknown. We further included *occupation* (white-collar worker [reference category], manual worker, manager/professional, self-employed, unemployed, retired, student and homemaker), and *marital status* (married or cohabitating [reference category], divorced or separated, single, widowed, and an undefined rest category “other”). A measure of the degree of urbanization of the place of residence indicates whether a respondent was living in a rural area or village (reference category), in a small or medium-sized town, or in a large town. Finally, at the individual level, we included an indicator of difficulty in finding information about mental health problems and how to cope with them (very easy = 1, very difficult = 4). Because 19% of the respondents answered “don't know,” we created a five-category dummy variable, including “don't know” and taking “very easy” as the reference category.

#### Country-Level Variables

For each country, we averaged scores on personal stigma beliefs to indicate country-specific *cultural stigma beliefs*. Calculation of the means of individual-level responses to obtain a group variable referring to culture is the most common aggregation strategy (Hofstede et al., 1990; Van Houtte, 2006). Following Klein and Kozlowski, collective properties that emerge through composition can be regarded as shared properties. Analogous strategies have been used in studies by Mojtabai (2010), by Evans-Lacko et al. (2012), and by Pachankis et al. (2014). Analysis of the variance components on the weighted sample indicates that a substantial share (8.3%) of the variance in personal stigma beliefs is between countries (SS_between countries_/(SS_between countries_ + SS_within countries_) = 0.032/(0.032 + 0.355), Chi^2^df = 27, *p* < 0.001).

In line with other studies, we measure supply-side effects using area provider-density scores (Scott and Shiell, 1997; Busato and Kunzi, 2008), derived from the WHO Statistical Information System (Organization World Health, 2009) and the World Mental Health Atlas 2005 (Organization World Health, 2005). We included the *number of psychiatrists and psychologists* per 100,000 inhabitants as an indicator of the supply of specialized mental health professionals, and the *number of general practitioners* per 100,000 inhabitants as an indicator of the availability of general health professionals.

### Analyses

To account for the individuals-within-countries sample design and the multi-level research problem (Hox, 2002), we estimated hierarchical linear models using MLwiN version 2.35. The dichotomous character of the outcomes justifies the use of logistic multi-level regression analysis. The MCMC approach was applied as well, given existing evidence that it can enhance the reliability of cross-level interaction terms in nesting structures containing low numbers of countries (Stegmueller, 2013). Models were run for 20,000 iterations in order to ensure convergence of the parameter estimates. All variables were centered on the grand mean, except for personal stigma beliefs, which was centered on the group mean, given that cultural stigma beliefs were added to the equation at the country level. The significance of the error terms measuring the randomness of the within-country slope of mental health complaints was taken into account. Non-response weights were used at the individual level. At the second level, countries were weighted to correct for different population sizes, thereby ensuring that each country is assigned the same importance in the multi-level regression analyses. Results are presented as odd ratios (ORs) for outcomes with a Bernoulli distribution, along with their accompanying 95% confidence intervals (CIs). Finally, the deviance information criterion (DIC) provides an indicator of the overall goodness of the model fit, with lower scores indicating a better model.

## Results

### Descriptive Statistics

With an overall mean of 2.18, European respondents generally did not agree with the items referring to stereotypes of dangerousness, unpredictability, responsibility, and poor prognosis (Appendix 1). High mean levels of negative stereotypes did appear among respondents in Latvia, Lithuania, Poland, and Italy, with lower levels observed for Luxembourg, the Netherlands, Ireland, and Spain. Within-country standard deviations ranged from 0.49 to 0.78 (no table), indicating substantial differences between individuals with regard to stigma beliefs. More important is the difference of 0.63 in cultural stigma beliefs between the countries with the highest (2.56) and lowest (1.93) mean levels of stereotypical beliefs, which is roughly consistent with the aforementioned standard deviations. This finding provides a crude indication of the importance of between-country variations in cultural stigma beliefs.

One important issue concerns whether our country-level stigma indicator can be regarded as a measure of cultural stigma beliefs. Only personal beliefs that are shared to a sufficient extent can be aggregated to a higher level in order to represent shared or cultural beliefs (Glick, 1985). One test that has been prescribed for this purpose is the intra-class correlation based on one-way analysis of variance: ICC (1, k) = (Between Mean Square-Within Mean Square)/Between Mean Square (Shrout and Fleiss, 1979; Glick, 1985), which should be higher than 0.60 (Glick, 1985). In our study, the ICC value is 0.90, which indicates justifies aggregating the measure of stereotypes to the country level, as they are sufficiently shared by the citizens of individual countries.

Substantial between-country differences also exist with regard to the prevalence of symptoms relating to depression and anxiety. High mean scores were found for countries some countries (e.g., Italy, Turkey, and Bulgaria), with lower rates in Denmark, Finland, Sweden, and Luxembourg. The table in Appendix 1 also reflects substantial cross-national variations in seeking support from either specialists or from generalists. Rates of self-reported help-seeking from GPs were low in Turkey, Malta, Cyprus, and Spain, with high rates in Romania and Slovakia. Low rates of consulting specialists were reported in Bulgaria and Romania, while high rates were observed in the Netherlands, France, Finland, Portugal, and Spain.

### Hierarchical Linear Models

Two models were estimated for each dependent variable. The first model includes all direct effects and explores between-country variations concerning the link between need and service use. The second model adds an interaction term to estimate whether the presence of outspoken cultural stigma beliefs moderates the association between need and the utilization of services (Table 1).

**Table 1:** Individual and country-level determinants of the utilization of mental health services from general practitioners and specialists (MLwiN logistic regression): odds ratios and 95% confidence intervals.

	**GP care**						**Specialist care**					
	**O.R**.	**C.I**.	**sig**	**O.R**.	**C.I**.	**sig**	**O.R**.	**C.I**.	**sig**	**O.R**.	**C.I**.	**sig**
Intercept	0.06	(0.05–0.08)	^***^	0.06	(0.05–0.09)	^***^	0.02	(0.01–0.03)	^***^	0.02	(0.01–0.03)	^***^
**INDIVIDUAL LEVEL**												
Personal stigma beliefs	0.82	(0.76–0.89)	^***^	0.82	(0.76–0.88)	^***^	0.51	(0.45–0.58)	^***^	0.51	(0.45–0.58)	^***^
Poor mental health	1.21	(1.19–1.24)	^***^	1.21	(1.19–1.24)	^***^	1.32	(1.29–1.35)	^***^	1.32	(1.29–1.35)	^***^
Gender	1.17	(1.06–1.29)	^**^	1.17	(1.07–1.29)	^**^	1.02	(0.88–1.19)		1.03	(0.89–1.20)	
Age	1.02	(1.01–1.02)	^***^	1.02	(0.99–1.02)	^***^	0.98	(0.97–0.98)	^***^	0.98	(0.97–0.98)	^***^
**Marital status (married/cohabit** **=** **ref)**												
Divorced	1.08	(0.94–1.23)		1.07	(0.93–1.23)		1.63	(1.35–1.96)	^***^	1.62	(1.35–1.95)	^***^
Single	0.98	(0.83–1.17)		0.99	(0.83–1.17)		0.96	(0.76–1.22)		0.97	(0.76–1.23)	
Widowed	0.95	(0.83–1.09)		0.94	(0.82–1.08)		0.76	(0.58–0.98)	^*^	0.75	(0.57–0.98)	^*^
Other	1.29	(0.77–2.16)		1.30	(0.76–2.17)		0.66	(0.27–1.65)		0.66	(0.27–1.61)	
**Education (< 16** **=** **ref)**												
16–19	1.02	(0.92–1.16)		1.02	(0.91–1.14)		1.01	(0.83–1.23)		1.01	(0.82–1.23)	
20+	0.92	(0.80–1.07)		0.92	(0.80–1.06)		1.14	(0.90–1.44)		1.13	(0.89–1.42)	
Unknown	0.91	(0.67–1.24)		0.90	(0.66–1.22)		0.85	(0.48–1.52)		0.85	(0.47–1.52)	
**Occupation (white collar** **=** **ref)**												
Unemployed	1.15	(0.93–1.45)		1.14	(0.92–1.43)		1.13	(0.82–1.58)		1.12	(0.81–1.54)	
Retired	1.25	(1.03–1.53)	^*^	1.24	(1.03–1.50)	^*^	1.77	(1.29–2.42)	^***^	1.75	(1.28–2.38)	^***^
Homemaker	1.15	(0.94–1.42)		1.14	(0.92–1.39)		0.93	(0.66–1.30)		0.92	(0.66–1.27)	
Student	0.82	(0.61–1.17)		0.82	(0.59–1.14)		0.68	(0.43–1.07)		0.67	(0.43–1.05)	
Self–employed	1.11	(0.88–1.41)		1.10	(0.88–1.39)		0.90	(0.62–1.29)		0.88	(0.62–1.25)	
Manager	0.92	(0.74–1.15)		0.92	(0.74–1.14)		1.26	(0.93–1.71)		1.25	(0.92–1.70)	
Manual worker	0.95	(0.79–1.14)		0.94	(0.78–1.12)		0.77	(0.58–1.03)		0.76	(0.58–1.01)	
**Urbanization (rural or village** **=** **ref)**												
Small or medium–sized	1.12	(1.02–1.25)	^*^	1.13	(1.02–1.25)	^*^	1.17	(0.98–1.39)		1.17	(0.98–1.39)	
town												
Large town	1.05	(0.94–1.18)		1.05	(0.94–1.18)		1.37	(1.14–1.64)	^***^	1.36	(1.14–1.63)	^***^
**Difficulty finding information (very easy** **=** **ref)**												
Easy	1.14	(0.98–1.32)		1.13	(0.98–1.30)		0.81	(0.67–0.98)	^*^	0.81	(0.67–0.98)	^*^
Difficult	1.21	(1.03–1.41)	^*^	1.21	(1.03–1.40)	^*^	0.73	(0.59–0.91)	^**^	0.73	(0.59–0.90)	^**^
Very difficult	1.20	(0.97–1.45)		1.19	(0.98–1.44)		0.66	(0.49–0.88)	^**^	0.66	(0.49–0.87)	^**^
Don't know	0.62	(0.52–0.75)	^***^	0.62	(0.52–0.74)	^***^	0.17	(0.12–0.25)	^***^	0.17	(0.12–0.25)	^***^
**COUNTRY LEVEL**												
Cultural stigma beliefs	0.62	(0.20–1.89)		0.72	(0.24–2.16)		0.15	(0.24–2.16)	^***^	0.15	(0.24–2.16)	^***^
Cultural stigma beliefs*Poor mental health				0.89	(0.80–0.98)	^*^				0.99	(0.88–1.13)	
Supply of GPs	0.99	(1.00–1.00)		0.99	(1.00–1.00)							
Supply of specialists							1.04	(1.01–1.07)	^*^	1.04	(1.01–1.07)	^*^
**VARIANCE**												
Intercept	0.30			0.3			0.22			0.22		
Random slope: MIH	0.01			0.01			0.01			0.01		
**DIC**	14173.5			14150.2			6441.6			6441.4		

* p < 0.05;

** p < 0.01;

*** *p < 0.001*.

First, the results support the need-based nature of the utilization of mental health services, as the latter is strongly associated with reports of mental health problems. Stronger effects emerged for seeking help from specialized professionals (OR = 1.32 [CI 95 %: 1.29, 1.35]) than for contacting GPs (OR = 1.21 [CI 95 %: 1.19, 1.24]). Second, personal stigma beliefs acted as barriers to both seeking specialized professional care (OR = 0.51 [CI 95 %: 0.45, 0.58]) and contacting GPs (OR = 0.82 [CI 95 %: 0.76, 0.89]). In this case as well, the association differed between generalized and specialized services. Neither of these findings is surprising, and, regardless of their substantive interpretation, they provide an indication of the convergent validity of the core variables. Third, in countries with strong cultural stigma beliefs, people avoid seeking support from specialists (OR = 0.15 [CI 95 %: 0.05, 0.46]), regardless of their personal beliefs. Moreover, these cultural effects are more pronounced than are the effects of personal beliefs. Fourth, cultural beliefs apparently did not inhibit respondents from consulting GPs for mental health problems in general (OR = 0.62 [CI 95 %: 0.20, 1.89]), although individuals with more severe mental health complaints apparently did refrain from consulting GPs in countries with high levels of cultural stigma beliefs, as indicated by the significant cross-level interaction effect (OR = 0.89 [CI 95 %: 0.80, 0.98]). No such cross-level interaction effect was identified for the utilization of specialized services.

The results further reveal that the supply of specialized services affects the likelihood of seeking support from specialists at the country level (OR = 1.04 [CI 95 %: 1.01, 1.07]), as well as at the level of within-country differences between rural regions and large towns (OR = 1.37 [CI 95 %: 1.14, 1.63]). While the availability of GPs seems irrelevant, service-supply effects on contacting GPs are apparent only between rural regions and small or medium-sized towns (OR = 1.12 [CI 95 %: 1.02, 1.25]), probably due to the high density of GPs in all regions except for truly rural areas.

The following differences emerged at the individual level. Regardless of their mental health status, women were more frequent users of general services, and elderly respondents had more contact with GPs, but less with specialists. In general, retired respondents used more services than other respondents did. Divorced respondents were more likely to utilize specialized professional care. The results do not reveal any educational differences.

Finally, the respondents who reported having difficulty finding information were less likely to consult specialists. In addition, those indicating that they did not know whether they had difficulty finding information were less likely to contact professionals. Additional analyses (no table) indicate that respondents in this sub-group hold more stigmatizing beliefs, and we suspect that their responses reflect either concealing behavior or disinterest in the subject.

Additional analyses also reveal that mental health problems were reported more frequently in countries with strong cultural stigma beliefs (No table: OR = 16.70 [7.03−40.10]) and in countries with few specialized mental health professionals (No table: OR = 0.94 [0.91−0.96]).

## Discussion and Conclusion

Using data from a broad set of European countries in a multi-level research design, we investigated the impact of both personal and cultural stigma beliefs on the likelihood of consulting GPs and specialized mental health professionals. The results indicate that contacting health professionals for mental health problems is related to the negative stereotypes that respondents held with regard to individuals with mental illness. In addition, and regardless of personal stigma beliefs, the dominant cultural beliefs proved relevant as well. Before discussing the findings, we summarize several important limitations.

First, the temporal ordering of the variables is not consistent with the presumed direction of causation: the utilization of mental health services is measured retrospectively, while mental health complaints and stigma beliefs are measured in the present. While this is common practice, it is obvious that the results could be distorted by recall bias and by processes of reverse causation. Contact with health professionals might generate negative stereotypes about individuals with mental illness. Second, several variables provide only rough indications of the underlying constructs. Mental health status is measured using a very short scale with high validity and reliability values, but limited to depression and anxiety, thereby ignoring other psychiatric complaints. While depression and anxiety are indeed the most common mental health problems, the measure is limited, and it probably underestimates the need for professional support. Despite these drawbacks, no other currently available international data contain information on all of the core variables in the present study. Similar limitations apply to the indicator of stigma beliefs, which addresses only four negative stereotypes of mental illness, with stereotypes being but one of the many aspects of stigma. They are nevertheless crucial, given that the stigma process starts with stereotypes (Corrigan and Watson, 2002; Angermeyer and Matschinger, 2005). Furthermore, the stigma items refer to “psychological or emotional health problems,” without using the term “mental illness” or referring to highly stigmatized diagnostic labels (e.g., schizophrenia). This could explain the generally low mean levels of negative stereotypes, and it probably reflect an underestimation of their occurrence. Moreover, the self-reported levels of stigma beliefs could be subject to social desirability bias, which could also limit cross-national comparability. Another drawback is the lack of information on income, which could have a substantial impact on the likelihood of seeking professional care (either general or specialized) for mental health problems in some countries (Sareen et al., 2007). We did include indicators of employment status and education. Furthermore, only very crude information was available on the supply of GPs and more specialized mental health professionals at the country level. The importance of supply-side factors in determining the likelihood of seeking professional help is thus almost certainly underestimated. Finally, we were able to use the information from 28 European countries provided by Eurostat. This is currently the broadest dataset of individuals in a sufficient number of European countries that provides information on stigma, mental health complaints, and the utilization of professional mental health services. Despite the substantial number of countries included, however, only a limited number of country-level control variables could be included. We were unable to extend the analyses to regions within countries as done by Beck et al. (2003), due to the very low numbers of respondents in most of the regions who had actually used these services.

One of our most important findings is that citizens of countries characterized by widespread negative stereotypes about individuals with mental health problems are less likely to utilize specialized mental health services, regardless of their personal stigma beliefs. According to our results, the dominant cultural stigma beliefs of a country affect the behavior of its citizens, independent of their own beliefs. This finding supports the notion that stigma is a cultural phenomenon (Phelan et al., 2014). The likelihood of consulting a GP for mental health problems was associated only with personal stigma beliefs, except among individuals with frequent mental health complaints. When interpreting these findings, it is important to consider these differential impacts of personal and cultural stigma beliefs, the difference between generalized and specialized care, and the fact that our indicator of poor mental health is limited to complaints relating to depression and anxiety.

First, it is important to note that personal stigma beliefs act as important barriers to seeking formal help, regardless of the impact of cultural stigma beliefs. Vogel et al. (2007) also demonstrate how the effect of public stigma is mediated by self-stigma, and Schomerus et al. (2009) report that only personal stigma perceptions—and not general stigma expectations—are related to the likelihood of seeking specialized mental health care for depression. In addition, both personal stigma beliefs and mental health problems are more strongly associated with the likelihood of seeking help from specialized professionals than from generalists. In line with findings reported in other studies, our results indicate that stigma poses much less of a barrier to consulting GPs in comparison with specialists (Barney et al., 2006), with people contacting specialized services only when their complaints are severe or when they have not internalized the prevailing cultural stigma beliefs. These results suggest that people wait too long before contacting specialized mental health services. They tend to turn to GPs for mental health problems at an earlier stage. This is favorable to the extent that GPs are capable of helping when mental health problems are involved. In many cases, however, the clients of GPs ultimately receive only pharmacological treatment (Lockhart and Guthrie, 2011; Mojtabai and Olfson, 2014), which is considered inadequate as a sole treatment.

It is important to note that these results apply only in countries whose cultures are less stigmatizing. In countries characterized by cultures of strong stigma beliefs, individuals with more severe mental health issues are also more likely to refrain from contacting GPs as well. We did not observe any similar cross-level interaction effect for the likelihood of seeking specialized care.

A core finding of this study is that, in countries with more outspoken cultural stigma beliefs, people encounter more barriers to seeking help from specialized mental health professionals, regardless of their personal beliefs. In these countries, people with more severe levels of psychiatric complaints also refrain from contacting general practitioners. Cultural stigma beliefs are thus associated with less utilization of specialized mental health services at all symptom levels, although only those with high levels of complaints are hesitant to make use of even generalized services. We would like to stress that the overall impact of cultural stigma is independent of personal stigma beliefs and the mental health of population. This is a relatively conservative observation, as we know that, on average, citizens in countries with strong cultural stigma beliefs tend to adhere to stronger personal stigma beliefs and to have poorer mental health.

In the absence of further information, we can only speculate on the mediating paths that link personal and cultural stigma beliefs to the utilization of generalized and specialized mental health services. It is generally acknowledged that individuals need not adhere to shared negative stereotypes personally in order to feel their impact. Even if they conceal their stigmatized identities, cultural stigma could lead to deterioration in their mental (or general) health (Quinn and Chaudoir, 2009). In highly stigmatizing cultures, expectations of devaluation and discrimination could lead to secrecy, avoidance, and withdrawal, for fear of negative societal reactions (Link et al., 1989), in addition to having an impact on the likelihood of seeking formal care. Such situations could lead individuals in need to postpone contacting professionals. In stigmatizing cultures, this is not limited to contacting psychiatrists, psychologists, or psychotherapists, but it could also discourage people from seeking help from general practitioners, who are contacted only in case of great need. Given that health professionals are also part of the shared culture, it is quite possible that, in highly stigmatizing cultures, people in need refrain from contacting them for fear that the professionals will be judgmental or disrespectful (Clement et al., 2015). In a meta-analysis of empirical studies, Clement et al. (2015) also identifies fear of discrimination, social rejection, social judgment, ridiculing, and lack of understanding as being among the dangers facing individuals who seek professional help in cultures characterized by a high level of public stigma. The lack of specialized mental health professionals or the lack of information about mental health issues can have a measurable impact on the likelihood of seeking help from specialists, although neither constitutes an important mediating path between cultural stigma beliefs and the likelihood of seeking specialized professional care, as both of these factors were taken into account in the analyses.

Given the differences that we have identified between service providers, future research on the link between need, stigma, and the utilization of mental health services should pay more attention to the type of services. The role of other health care providers (e.g., nurses and alternative and complementary health service providers) or other forms of care or support that provide less stigmatizing forms of non-clinical care (Clement et al., 2015) (e.g., social workers and community based initiatives) should be considered. Given their function as gatekeepers to more specialized care, the attitudes of GPs with regard to mental illness and specialized mental health care deserve more attention as well (Podgorski and Caine, 1996; Goldfracht et al., 2007). It is plausible that, in countries with a higher prevalence of stigma beliefs, GPs might have an even stronger tendency to rely uncritically on biomedical frameworks in order to understand mental health problems. They might therefore be more inclined to refer their patients to more specialized psychiatric care or to initiate pharmacological treatment on their own. In some countries, GPs prescribe more than 60% of all anti-depressants and anxiolytics (Mark et al., 2009), and their impact is increasing (Olfson et al., 2014). This strategy has proven less than optimal, and even harmful for most individuals with common mental health problems (Gøtzsche et al., 2015). Overall, the finding concerning the impact of a shared, cultural, stigma effect on the likelihood of seeking professional care regardless of personal stigma beliefs suggests that stigma constitutes a population-wide health problem (Hatzenbuehler et al., 2013) that requires intervention aimed at the more fundamental social causes of stigma. Finally, it is important to note that cultural stigma beliefs involve entire populations, while personal stigma beliefs have a more limited impact on the individuals who are affected and their significant others (e.g., family members, co-workers, and friends).

In conclusion, the multi-level, cross-national comparative research design of the present study makes it possible to disentangle the individual and cultural effects of stigma on the actual utilization of services. We can confirm that the dominant cultural orientation toward mental health problems poses a true barrier to seeking professional help, regardless of individual stigma beliefs. Given that the majority of studies on stigma and the seeking of professional care ignores this cultural dimension of stigma, they are likely to underestimate the devastating consequences of stigma beliefs. Because cultural stigma beliefs are a societal feature, our research provides further evidence that stigma might pose an important threat, both to individual health and to public health in general (Link and Phelan, 2006). The effects of stigma beliefs that are embedded within a common culture are not limited to the effectiveness of specific mental health services (Rosenfield, 1997; Sirey et al., 2001; Verhaeghe et al., 2010; Verhaeghe and Bracke, 2012). They are also likely to affect the mental health of the population as a whole, as stigma acts as a barrier to professional mental health support for individuals who are currently in need of such support, as well as for those who might need it in the future. Addressing the adverse effects of stigma could thus be beneficial to both those who are stigmatized and those who engage in stigmatizing.

## Author Contributions

All authors listed have made a substantial, direct and intellectual contribution to the work, and approved it for publication.

### Conflict of Interest Statement

The authors declare that the research was conducted in the absence of any commercial or financial relationships that could be construed as a potential conflict of interest.
