# Supplementary material for: Dominant Cultural and Personal Stigma Beliefs and the Utilization of Mental Health Services: A Cross-National Comparison
Source: Front Sociol. 2019 May 8;4:40. doi: 10.3389/fsoc.2019.00040 (PMC8022809; doi:10.3389/fsoc.2019.00040)
Supplement: Supplementary file 1 [file Table_1.docx]

| Appendix 1. Individual-level variables (average scores per country) and country-level variables (weighted sample) | | | | | | | | | | | | | | | |  |
| --- | --- | --- | --- | --- | --- | --- | --- | --- | --- | --- | --- | --- | --- | --- | --- | --- |
| Country | Unweighted sample size | | Consult GP | Consult specialist | Stigma beliefs | Depression  /anxiety | Age | Women | Married/ cohab. | Education (<16y) | White collar | Rural or village | Finding information very easy | GP supply (/100,000) | Specialist supply (/100,000) | |
| **C-E Europe** | |  |  |  |  |  |  |  |  |  |  |  |  |  |  | |
| Austria | | 890 | 0.15 | 0.02 | 2.05 | 11.0 | 47.8 | 0.54 | 0.58 | 0.36 | 0.09 | 0.48 | 0.08 | 370 | 11.8 | |
| Bulgaria | | 782 | 0.16 | 0.01 | 2.34 | 12.2 | 48.5 | 0.54 | 0.65 | 0.22 | 0.07 | 0.29 | 0.09 | 30 | 9.00 | |
| Czech Republic | | 914 | 0.10 | 0.04 | 2.16 | 10.8 | 47.1 | 0.52 | 0.54 | 0.12 | 0.21 | 0.27 | 0.16 | 360 | 12.1 | |
| Hungary | | 866 | 0.07 | 0.05 | 2.05 | 11.0 | 42.6 | 0.49 | 0.57 | 0.32 | 0.06 | 0.37 | 0.08 | 300 | 9.00 | |
| Latvia | | 881 | 0.07 | 0.02 | 2.56 | 11.9 | 40.9 | 0.49 | 0.57 | 0.16 | 0.08 | 0.33 | 0.13 | 310 | 10.00 | |
| Lithuania | | 878 | 0.11 | 0.03 | 2.56 | 11.6 | 43.0 | 0.48 | 0.61 | 0.16 | 0.08 | 0.23 | 0.11 | 400 | 15.00 | |
| Poland | | 850 | 0.08 | 0.04 | 2.43 | 11.3 | 43.6 | 0.52 | 0.62 | 0.21 | 0.09 | 0.31 | 0.12 | 200 | 6.00 | |
| Romania | | 773 | 0.20 | 0.01 | 2.31 | 11.6 | 43.7 | 0.47 | 0.65 | 0.25 | 0.09 | 0.42 | 0.08 | 310 | 4.10 | |
| Slovakia | | 905 | 0.15 | 0.03 | 2.25 | 10.7 | 41.0 | 0.49 | 0.49 | 0.17 | 0.13 | 0.51 | 0.10 | 240 | 10.00 | |
| Slovenia | | 948 | 0.10 | 0.03 | 2.18 | 11.0 | 47.2 | 0.48 | 0.64 | 0.29 | 0.11 | 0.41 | 0.11 | 190 | 5.35 | |
| **Mediterranean Europe** | | | |  |  |  |  |  |  |  |  |  |  |  |  | |
| Croatia | | 912 | 0.10 | 0.04 | 2.40 | 11.8 | 47.4 | 0.47 | 0.58 | 0.31 | 0.08 | 0.37 | 0.14 | 250 | 8.70 | |
| Cyprus | | 900 | 0.03 | 0.04 | 2.16 | 11.9 | 44.3 | 0.48 | 0.69 | 0.43 | 0.16 | 0.31 | 0.32 | 230 | 5.00 | |
| Greece | | 959 | 0.06 | 0.03 | 2.32 | 11.7 | 44.7 | 0.42 | 0.59 | 0.43 | 0.09 | 0.30 | 0.33 | 500 | 15.00 | |
| Italy | | 898 | 0.10 | 0.02 | 2.48 | 13.0 | 54.5 | 0.52 | 0.53 | 0.48 | 0.12 | 0.17 | 0.07 | 370 | 9.80 | |
| Malta | | 408 | 0.05 | 0.02 | 2.14 | 10.6 | 41.5 | 0.34 | 0.73 | 0.28 | 0.10 | 0.30 | 0.12 | 390 | 4.00 | |
| Portugal | | 840 | 0.10 | 0.06 | 2.13 | 11.3 | 42.6 | 0.47 | 0.68 | 0.62 | 0.08 | 0.72 | 0.06 | 340 | 4.70 | |
| Spain | | 860 | 0.06 | 0.06 | 1.93 | 10.7 | 45.1 | 0.41 | 0.62 | 0.40 | 0.13 | 0.39 | 0.16 | 330 | 3.60 | |
| Turkey | | 836 | 0.04 | 0.04 | 2.31 | 12.3 | 37.4 | 0.52 | 0.65 | 0.69 | 0.03 | 0.48 | 0.16 | 160 | 1.00 | |
| **N-W Europe** | |  |  |  |  |  |  |  |  |  |  |  |  |  |  | |
| Belgium | | 937 | 0.08 | 0.04 | 2.12 | 10.4 | 50.2 | 0.51 | 0.54 | 0.20 | 0.13 | 0.50 | 0.14 | 420 | 18.00 | |
| Denmark | | 953 | 0.13 | 0.05 | 2.01 | 9.5 | 46.0 | 0.54 | 0.54 | 0.17 | 0.09 | 0.26 | 0.18 | 360 | 16.00 | |
| Finland | | 952 | 0.06 | 0.06 | 2.02 | 9.6 | 44.7 | 0.44 | 0.54 | 0.20 | 0.06 | 0.28 | 0.18 | 330 | 22.00 | |
| France | | 942 | 0.11 | 0.07 | 2.02 | 10.4 | 45.5 | 0.51 | 0.58 | 0.34 | 0.10 | 0.42 | 0.08 | 340 | 22.00 | |
| Germany | | 1372 | 0.12 | 0.04 | 2.02 | 10.9 | 48.3 | 0.5 | 0.62 | 0.3 | 0.07 | 0.35 | 0.17 | 340 | 11.80 | |
| Ireland | | 848 | 0.14 | 0.02 | 1.94 | 10.3 | 44.5 | 0.47 | 0.52 | 0.28 | 0.08 | 0.40 | 0.21 | 190 | 6.82 | |
| Luxembourg | | 446 | 0.14 | 0.04 | 1.93 | 9.4 | 42.1 | 0.44 | 0.65 | 0.22 | 0.22 | 0.51 | 0.08 | 270 | 12.00 | |
| Netherlands | | 1052 | 0.09 | 0.10 | 1.96 | 10.0 | 43.9 | 0.55 | 0.53 | 0.18 | 0.18 | 0.45 | 0.35 | 370 | 9.00 | |
| Sweden | | 942 | 0.08 | 0.04 | 2.08 | 9.4 | 48.3 | 0.54 | 0.48 | 0.15 | 0.15 | 0.45 | 0.12 | 440 | 20.00 | |
| UK & N. Ireland | | 1137 | 0.11 | 0.02 | 2.13 | 10.2 | 44.3 | 0.52 | 0.51 | 0.10 | 0.10 | 0.29 | 0.15 | 230 | 11.00 | |
| Total | | 24881 | 0.10 | 0.04 | 2.18 | 10.9 | 45.3 | 0.49 | 0.58 | 0.29 | 0.11 | 0.37 | 0.15 | 311.21 | 4.63 | |
